# Pyrethroid resistance and gene expression profile of a new resistant *An. gambiae* colony from Uganda reveals multiple resistance mechanisms and overexpression of Glutathione-S-Transferases linked to survival of PBO-pyrethroid combination

**DOI:** 10.12688/wellcomeopenres.19404.1

**Published:** 2024-01-08

**Authors:** Ambrose Oruni, Amy Lynd, Harun Njoroge, Ismail Onyige, Arjen E. van’t Hof, Enock Matovu, Martin J. Donnelly

**Affiliations:** 1 Department of Vector Biology, Liverpool School of Tropical Medicine, Liverpool, Merseyside, L3 5QA, UK; 2 College of Veterinary Medicine, Animal Resources and Biosecurity, Makerere University, Kampala, Central Region, Uganda; 3 Centre for Global Health Research, Kenya Medical Research Institute (KEMRI), Kisumu, Kenya; 4 Infectious Diseases Research Collaboration, Kampala, Central Region, Uganda

**Keywords:** Uganda, Mosquito colony, Anopheles gambiae, Pyrethroids, Insecticide resistance, Long‐lasting insecticidal nets (LLINs), Piperonyl butoxide (PBO), Bioassays, Gene expression, Glutathione-s-transferase (GST), Cytochrome P450s, Vector control

## Abstract

**Background:** The effectiveness of long-lasting insecticidal nets (LLINs) are being threatened by growing resistance to pyrethroids. To restore their efficacy, a synergist, piperonyl butoxide (PBO) which inhibits cytochrome P450s has been incorporated into pyrethroid treated nets. A trial of PBO-LLINs was conducted in Uganda from 2017 and we attempted to characterize mechanisms of resistance that could impact intervention efficacy.

**Methods:** We established an *Anopheles gambiae* s.s colony in 2018 using female mosquitoes collected from Busia district in eastern Uganda. We first assessed the phenotypic resistance profile of this colony using WHO tube and net assays using a deltamethrin dose-response approach. The Busia colony was screened for known resistance markers and RT-qPCR targeting 15 genes previously associated with insecticide resistance was performed.

**Results:** The Busia colony had very high resistance to deltamethrin, permethrin and DDT. In addition, the colony had moderate resistance to alpha-cypermethrin and lambda-cyhalothrin but were fully susceptible to bendiocarb and fenitrothion. Exposure to PBO in combination with permethrin and deltamethrin resulted in higher mortality rates in both net and tube assays, with a higher mortality observed in net assays than tube assays. The *kdr* marker, *Vgsc-995S* was at very high frequency (91.7-98.9%) whilst the metabolic markers *Coeae1d* and *Cyp4j5-L43F* were at very low (1.3% - 11.5%) and moderate (39.5% - 44.7%) frequencies respectively. Our analysis showed that gene expression pattern in mosquitoes exposed to deltamethrin, permethrin or DDT only were similar in comparison to the susceptible strain and there was significant overexpression of cytochrome P450s, glutathione-s-transferases (GSTs) and carboxyl esterases (COEs). However, mosquitoes exposed to both PBO and pyrethroid strikingly and significantly only overexpressed closely related GSTs compared to unexposed mosquitoes while major cytochrome P450s were underexpressed.

**Conclusions:** The high levels of pyrethroid resistance observed in Busia appears associated with a wide range of metabolic gene families.

## Introduction

Uganda has one of highest malaria burdens in sub-Saharan Africa ( WHO, 2018), with very high infection rates occurring in the eastern part of the country ( Rugnao *et al.*, 2019). To control malaria, vector control efforts have been scaled-up over the past 20 years, mostly through the wide distribution of pyrethroid insecticide treated bed-nets (ITNs), supplemented by indoor residual spraying (IRS) ( Katureebe *et al.*, 2016). However, the increase and spread in resistance to pyrethroids ( Lynd *et al.,* 2018; Mawejje *et al.,* 2013a; Ranson & Lissenden, 2016), the primary insecticide used on bed-nets ( WHO, 2016), is detrimental to the success of malaria control. Insecticide resistance is attributed to four main mechanisms: namely metabolic resistance, which occurs through increased expression or allelic variation in detoxifying genes that breakdown insecticides ( Hemingway *et al.*, 2004); target-site resistance, which occurs through polymorphisms that alter insecticide binding sites ( Donnelly *et al.*, 2016); as well as the less understood cuticular resistance and behavioral resistance. The high levels of pyrethroid resistance in Uganda ( Mawejje *et al.*, 2013a; Mulamba *et al.,* 2014b; Okia *et al.,* 2018) and indeed the rest of sub-Saharan Africa ( Lynd *et al.*, 2018; Ranson & Lissenden, 2016) is commonly associated with cytochrome P450 enzymes ( David *et al.,* 2013; Djouaka *et al.,* 2016; Mulamba *et al.,* 2014b; Pondeville *et al.,* 2013; Scott, 1999; Wondji *et al.,* 2009). This has prompted the deployment of second-generation long-lasting insecticidal nets (LLINs) in which a synergist, piperonyl butoxide (PBO), is incorporated with the pyrethroid insecticide ( WHO, 2017). The PBO acts by inhibiting the action of P450s, thus overcoming the metabolic resistance and restoring the killing effect of the pyrethroid ( Bingham *et al.*, 2011; Snoeck *et al.*, 2017). Therefore, PBO-nets could help to partially check the resistance mediated by cytochrome P450s hence aiding effective vector control. Indeed, recently it was observed that children (2-10yr old) from communities with PBO-nets had lower malaria infection prevalence compared to children from communities with standard, non-PBO nets ( Maiteki-Sebuguzi *et al.*, 2023; Staedke *et al.*, 2020). However, there has been an increasing trend in the intensity of metabolic resistance in several Anopheline mosquito vectors ( Lynd *et al.*, 2018; Njoroge *et al.*, 2022; Stica *et al.*, 2019a; Weetman *et al.*, 2018). There are reports of mosquitoes surviving PBO-pyrethroid exposure from a number of countries in Africa including Uganda ( Gleave *et al.*, 2021) but an understanding of the cause is lacking. Crucially, investigating the mechanisms by which mosquitoes overcome PBO exposure is vital for vector control and insecticide resistance management (IRM). There is already evidence for loss of efficacy to PBO nets against *An. funestus* attributed to both GSTs ( Menze *et al.*, 2020) and P450 duplications ( Mugenzi *et al.*, 2019) leading to successful blood-feeding by resistant mosquitoes.

In this study, we used a recently colonized *Anopheles gambiae s.s* line from Busia district in eastern Uganda to examine genetic variants potentially driving resistance with a focus on tolerance to PBO-pyrethroid combinations. The expression levels of 15 genes commonly associated with metabolic resistance in *An. gambiae* mosquitoes were investigated.

## Methods

### Ethics approval and consent to participate

The Wellcome Trust International Master’s Fellowship (203511) project study was incorporated within the PRISM-study (NIH/NIAID U19AI089674) and the PBO-net study (R01AI116811) approved by the Ugandan National Council for Science and Technology (UNCST Ref HS 2176, on 20/12/2016), and the Liverpool School of Tropical Medicine (Ref 16‐072, on 8/02/2017) which supported this study. Written informed consent to collect mosquitoes in the study was obtained from the head of household (or their designate) for all participating households.

### Field mosquito collection and species diagnostic PCR

Mosquitoes were collected from 12 households in Busia District, Uganda, within the South-Bugwere Health sub-District (HSD) (0°19'01.0"N 33°58'00.5"E) *.* Houses were selected from an ongoing cohort study based on data showing malaria cases reported within one year. From the top 12 houses on the list, household heads were approached, the aim of the study explained to them and their permission sought to collect mosquitoes from their houses. Once they consented, they were informed a day before the collection about the activities. Indoor-resting, blood-fed, female mosquitoes were collected using a Prokopack aspirator between 05:00 and 08:00 between 01 ^st^ October and 19 ^th^ November 2018. Mosquitoes were transported in a cool box to a nearby insectary and morphologically identified as *An. gambiae* s.l before being transferred into standard 30cm ^3^ BugDorm cages (Watkins & Doncaster, Leominster, UK). We used species diagnostic PCR ( Chabi *et al.*, 2019) to differentiate *An. gambiae s.s.* from *An. arabiensis* collected from the field and at G1 generation to assess colony composition.

### Establishment of the BusiaUG colony

A total of 384 female mosquitoes were collected, of which 150 gravid females were made to oviposit by forced egg laying method. Briefly, a gravid female about 5 days old is placed inside a 1.5ml Eppendorf tube containing moist filter paper. Three holes are then pierced at the top of the lid. Mosquitoes are then kept for 24 to 48 hours to allow oviposition ( Morgan *et al.*, 2010). The remaining females laid eggs by cage oviposition where a moist Whatman filter placed on an oviposition cup is placed inside the cage to allow oviposition. The resulting eggs were kept at 4-10 ^o^C prior to and during shipment to Liverpool where they were immediately transferred to hatching containers on arrival. Mosquitoes were reared following standard insectary protocols. Emerged adults were kept in a large polyester mesh net measuring 60x60x60 cm to improve mating success. Mosquitoes were then subdivided into groups of approximately 200 mosquitoes each at day-5 and kept in ten smaller cages measuring 20x20x20 cm in which they were also blood fed. Female mosquitoes were arm fed in the dark following an overnight starvation of 8 hours, and then maintained on 10% sucrose. A decrease in oviposition rates was observed from generation one (G1) to generation six (G6) which we attempted to overcome by biweekly blood feeds and multiple feeding attempts per day. Adults from different oviposition rounds but the same generation were pooled into single cages. Females were provided with a black cup for oviposition. This procedure was repeated for about 20 weeks until the colony was stable and a higher number of emerged adult mosquitoes (>200 mosquitoes) were obtained from a single oviposition. For this study, mosquitoes from up to 11 ^th^ generation (G11) which were unselected, were used. Insecticide resistance selection was performed at the 12 ^th^ generation (G12) using 0.05% Deltamethrin. The colony was named “BusiaUG” where “Busia” denoting the district where the mosquitoes were collected and “UG” denoting Uganda.

### Mosquito rearing conditions

 All *An. gambiae* s.l were reared under standard insectary conditions at temperature of between 25-28 ^o^C with 75-85% relative humidity under a 12:12 photoperiod. Two colonies (Tiassale and Kisumu) were used for comparative purposes. Tiassale was established in 2009 in Cote d’Ivoire and has been maintained under selection using pyrethroids at LSTM since 2013. It became resistant to pyrethroids and DDT by 2018 with only *Vgsc-995F kdr* genotype but low level of fenitrothion resistance with both the G119S and 119G *Ace-1* genotypes present. The Kisumu strain, from western Kenya, is susceptible to all pyrethroids and carbamates but exhibits some tolerance to DDT ( Williams *et al.*, 2019).

### WHO bioassays (resistance phenotyping)

For all resistance phenotyping, 3-5-day old females were used. To estimate natural levels of resistance G1 mosquitoes were exposed to LLINs using the WHO cone assay for 3 minutes, after which 1hr knockdown and 24hr mortality were recorded. In addition, G1 mosquitoes were exposed to discriminating dose of deltamethrin (0.05% - 1x) using the WHO tube assay ( WHO/GMP, 2016), and a concentration series (2x, 3x, 5x and 10x), to assess the level of resistance. After the colony establishment, mosquitoes from G9-G11 were exposed to standard WHO insecticide impregnated papers; 0.05% deltamethrin, 0.75% permethrin, 4% DDT, 0.05% cypermethrin, 0.05% cyhalothrin, 1% fenitrothion, 0.1% bendiocarb and PBO (4%) synergistic assays with deltamethrin or permethrin. All procedures were conducted according to WHO guidelines ( WHO/GMP, 2016). Mosquitoes were kept on 10% sucrose during the 24hr recovery period. Mosquitoes that survived insecticide exposure were kept at -80 ^o^C for RNA extraction, whilst dead mosquitoes were stored on silica gel for DNA extraction.

During exposure of mosquitoes to insecticides using WHO tube assays, a minimum of 2 (n=2) and maximum of 4 replicate experiments comprising of ≈25 alive mosquitoes were performed to obtain 5-10 pools of alive mosquitoes for each insecticide type for gene expression analysis. For deltamethrin, permethrin, DDT, PBO + deltamethrin, bendiocarb and fenitrothion, four replicates were used; for PBO + permethrin, three replicates) were used, and for alpha-cypermethrin and lambda-cyhalothrin, two replicates were used.

### Resistance genotyping

DNA was extracted from the mosquitoes using the nexttec™ 1-Step kit (Biotechnologie GmbH, Hilgertshausen, Germany) according to manufacturer’s instructions. Molecular identification was used to discriminate *An. gambiae* s.s. and *An. arabiensis* ( Chabi *et al.*, 2019) *.* Mosquitoes were genotyped for the common resistance markers; *Coeae1d*, *Cyp4j5* ( Weetman *et al.*, 2018) and *Vgsc*-L995F/S ( *Kdr*) ( Lynd *et al.*, 2018).

### RT-qPCR

RNA was extracted from pools of 5-10 mosquitoes which survived insecticide exposure. These groups were; live mosquitoes after exposure to insecticides; deltamethrin, permethrin, DDT, alpha-cypermethrin, lambda-cyhalothrin, PBO + permethrin, PBO + deltamethrin and PBO only (as experimental control) and unexposed mosquitoes from the BusiaUG colony, Kisumu (as susceptible control) and Tiassale (as resistant control). RNA was extracted from all the groups except Tiassale which was obtained from an earlier study ( Ingham *et al.,* 2018). RNA was extracted using the PicoPure® RNA Isolation Kit, (ThermoFisher scientific, United Kingdom) followed by cDNA synthesis using the SuperScript® III First-Strand Synthesis System, ThermoFisher (United Kingdom) and subsequent purification of the cDNA using a QIAquick® PCR Purification Kit (Qiagen). All three procedures were done according to manufacturers’ manual. Both RNA and cDNA were stored at -80 ^o^C.

Expression patterns of 15 potential metabolic resistance genes were investigated ( Edi *et al.,* 2014; Ingham *et al.,* 2018; Irving *et al.,* 2012; Mulamba *et al.,* 2014a; Stica *et al.,* 2019b; Wilding *et al.,* 2014). Primers for qPCR were re-designed using the DNA sequences from the Ag1000g database ( The Anopheles gambiae 1000 Genomes Consortium, 2017) to avoid mutations that could affect annealing (supplementary Table S1, Oruni, 2023d). Reactions were carried out in a final volume of 20 µl consisting of 1x Brilliant III Utra-Fast SYBR® mix (Agilent Technologies, United Kingdom), 300nM of each primer and 1.0µl of 1:10 diluted cDNA. The qPCR assay was performed on the AriaMX Real-time PCR System (Agilent technologies) with an initial denaturation at 95 ^o^C for 1 min, followed by 40 cycles of 95 ^o^C for 10s, 60 ^o^C for 30s). A final melt curve from 55 ^o^C to 95 ^o^C was performed to check amplification quality. Standard curves for all primer sets were carried out using Kisumu cDNA to determine primer efficiency. Cycle threshold (Cq) values were analyzed using the ΔΔct Pfaffl method ( Pfaffl, 1999) and comparative Ct method ( Schmittgen & Livak, 2008) using the RPS7 gene as an endogenous control for normalization of expression. Gene expression of BusiaUG survivors for each insecticide exposure were compared to the susceptible population (Kisumu) and the fold change reported.

For the RNA experiments, three biological replicates (n=3) were used, except for lambda-cyhalothrin and PBO + deltamethrin exposed groups where two biological replicates (n=2) were used. The variation in number of biological replicates was because only 12 live mosquitoes were obtained (giving 6 mosquitoes per replicate) from PBO-deltamethrin exposure, while for lambda-cyhalothrin, one of the replicates had very low RNA concentration and was discarded. However, in all the RNA experiments, three technical replicates (n=3) were used. All primer pairs, except for the *Cyp4c28* gene (with efficiency of 76.3% and hence not discussed further) were within the acceptable efficiency range of 90–120%.

### Gene expression analysis

We used Ct-values to calculate relative fold change. The resistant population comprised of the BusiaUG treatment groups (exposed, unexposed) and unexposed Tiassale, while the reference control susceptible population was the Kisumu laboratory strain. Using the unexposed group as the baseline, we calculated relative fold change expression for all exposed groups. Genes that were overexpressed and those that were downregulated in resistant population of *An. gambiae s.s* from eastern Uganda following exposure to pyrethroids or PBO were identified. The mean fold change of gene expression for all groups was visualised using heatmaply package on R software. ANOVA was used to calculate the significant differences in mean fold changes between the exposed and unexposed groups, with a threshold at P=0.05. To further investigate the gene expression pattern observed in mosquitoes exposed to PBO, a STRING network analysis ( Szklarczyk *et al.*, 2019) was used to identify the relationship between the genes.

## Results

### *An. gambaie* s.l species composition

Of the 348 mosquitoes obtained in the first household collections for preliminary assessment of field resistance, 95% were *An. gambiae s.s* and 3.4% *An. Arabiensis* ( Oruni, 2023a & Oruni, 2023b). A total of 384 mosquitoes were obtained in the second collection for colony establishment, of which 97.7% and 2.3% were *An. gambiae s.s* and *An. arabiensis* respectively. During colony establishment the collections were mixed, although all *An. arabiensis* were lost at G1 such that 100% of the G2 population were *An. gambiae s.s.*

### Phenotypic resistance profile

**Figure 1.**
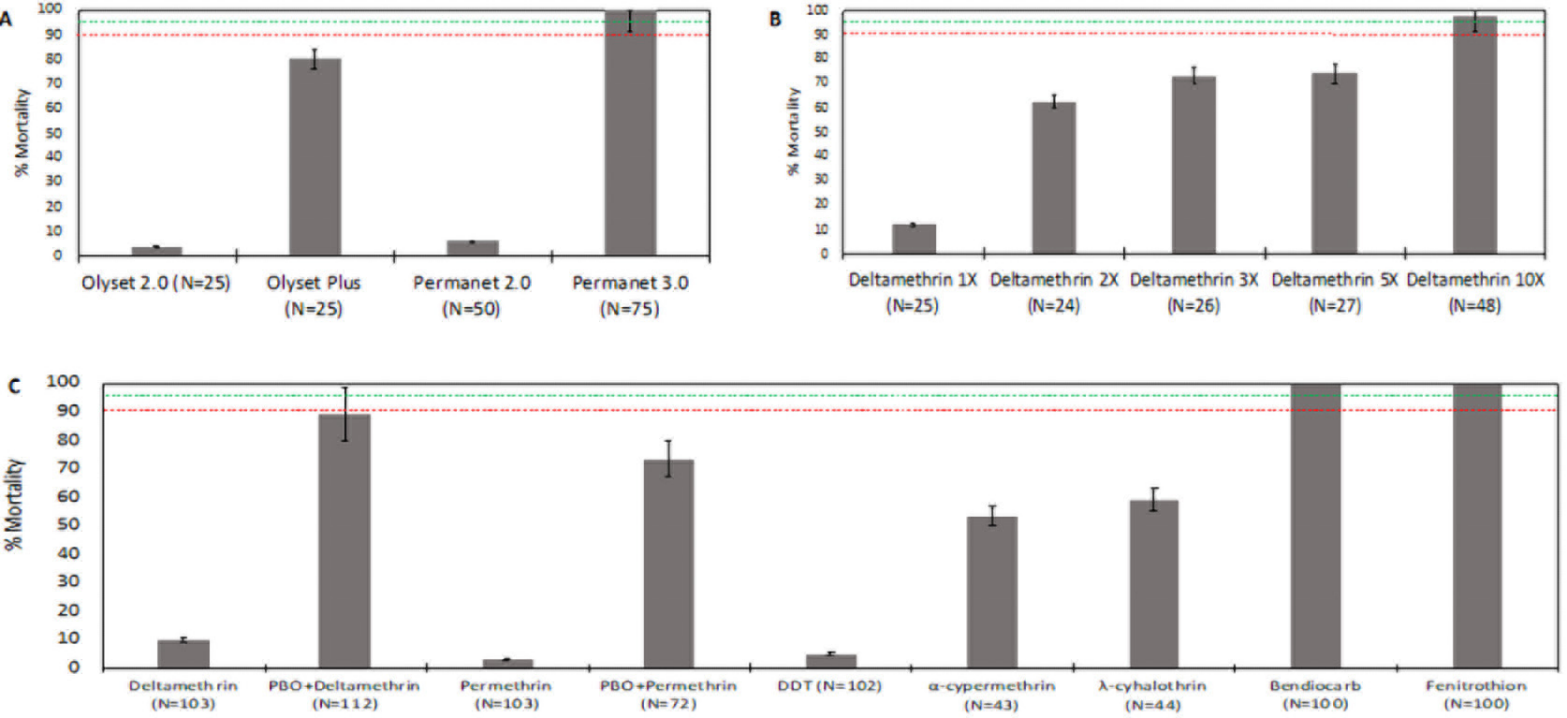
Resistance profile of *An. gambiae s.s* mosquitoes from the field and BusiaUG colony following exposure insecticides. Female mosquitoes from Busia exhibited very high resistance (low % mortality) when exposed to pyrethroids without piperonyl butoxide (PBO) by both field WHO net assays (permethrin-Olyset 2.0 or deltamethrin-PermaNet 3.0) (panel **A**) and WHO tube assays on the colony at G1 (panel **B**) or G9 (panel **C**). Colony female mosquitoes also showed high intensity of resistance by surviving deltamethrin at 5x and 10x with increased mortality at higher doses ( **B**). Cypermethrin and cyhalothrin showed moderate resistance and there was full susceptibility to organophosphates and carbamates ( **C**). The mosquito mortality by pyrethroids greatly increased when females were exposed in presence of a cytochrome P450 inhibitor, PBO, both with the WHO net assays using Olyset Plus and PermaNet 3.0 top ( **A**) and WHO synergistic tube assays ( **C**). Higher mortality was observed with WHO net assays using PBO nets compared to WHO tube assays especially with PermaNet 3.0 but not with Olyset Plus. Dotted lines indicate resistance levels where above 98% (green) it’s susceptible while below 90% (red) is confirmed resistance. Between 98% and 90% is suspected resistance. The error bars are SEM at 95% confidence interval.

Female mosquitoes collected from the field and BuisaUG colony at generation one (G1) and nine (G9) were exposed to an array of insecticides. Female mosquitoes exposed to only pyrethroids commonly used in LLINs i.e. deltamethrin and permethrin, or DDT showed very high resistance both in the field and from the colony. WHO cone assays were only done using G1 mosquitoes which showed very low mortality to pyrethroid-only nets; PermaNet 2.0 (6.0%, 95% CI; 5.6-6.4) and Olyset Net (4.0%, 95% CI; 3.8-4.2; 95%) ( Figure 1A). WHO tube assays also caused low mortality with deltamethrin, permethrin and DDT impregnated papers ( Figure 1C). In addition, exposure to 5x and 10x the discriminatory dose did not result in complete mortality ( Figure 1B). Full susceptibility (100% mortality) was only observed with carbamates and organophosphates in WHO tube assays ( Figure 1C).

Exposure of mosquitoes to PBO using either PBO-net cone assays, or pre-exposure in WHO tube assays significantly increased mortality to permethrin and deltamethrin, suggesting a role for cytochrome P450s in resistance. In cone assays against dual LLINs, full susceptibility was observed against the top surface of PermaNet 3.0 (deltamethrin + PBO), whilst 80.0% (95% CI; 76.1-83.9; 95% CI) mortality was observed against Olyset Plus (permethrin + PBO) ( Figure 1A). A similar range of mortality was observed with WHO synergistic assays ( Figure 1C).

### Frequency of resistance markers

Markers previously associated with resistance in East Africa ( Lynd *et al.*, 2018) were assessed in field collected mosquitoes and in G1 mosquitoes of the BusiaUG colony. All three genotypes of the mutant kdr alleles (serine (S), phenylalanine (F) and heterozygote (FS)) were present at the Vgsc-995 locus in the field population. The allele frequency of *Vgsc-995S* allele was 87.5% in *An. gambiae s.s.* and 4.2% in *An. arabiensis* and that of the *Vgsc-995F* was 3.13% all from *An. arabiensis* based on the sampled mosquitoes. All resistance genotypes for the metabolic marker *Cyp4j5-L43F* and *Coeae1d* variant were confirmed present at 1.3%, 78.9% and 19.7% for mutant allele, heterozygote and wild-type allele for *Cyp4j5-43F* and 44.7%, 43.4% and 14.5% for *Coeae1d* ( Table 1).

**Table 1.** Allele frequency of resistance markers from *An. gambiae s.s* mosquitoes sampled from the field and BusiaUG colony. RR represents the homozygous allele for resistance, RS represents the heterozygous allele and SS represents the homozygous allele for susceptibility.

Test sample	Marker	N	Frequency		
			RR	RS	SS
*G1 from the field*	*Vgsc-*L995S	96	88(91.7%)	0 (0.0%)	0 (0.0%)
	*Vgsc-*L *995F*	96	3 (3.13%)	5(5.2%)	0 (0.0%)
	*Cyp4j5*-L43F	92	11 (11.5%)	58 (60.4%)	23 (24.0%)
	*Coeae1d*	95	38(39.5%)	32(33.3%)	25(26.0%)
*G1 from the BusiaUG colony*	*Vgsc-*L995S	90	89 (98.9%)	0 (0.0%)	1(1.1%)
	*Vgsc-*L995SF	90	0 (0.0%)	0 (0.0%)	0 (0.0%)
	*Cyp4j5*-L43F	76	1 (1.3%)	60 (78.9%)	15 (19.7%)
	*Coeae1d*	78	34(44.7%)	33(43.4%)	11(14.5%)

### Transcriptomic (Gene expression) profiles

**Figure 2.**
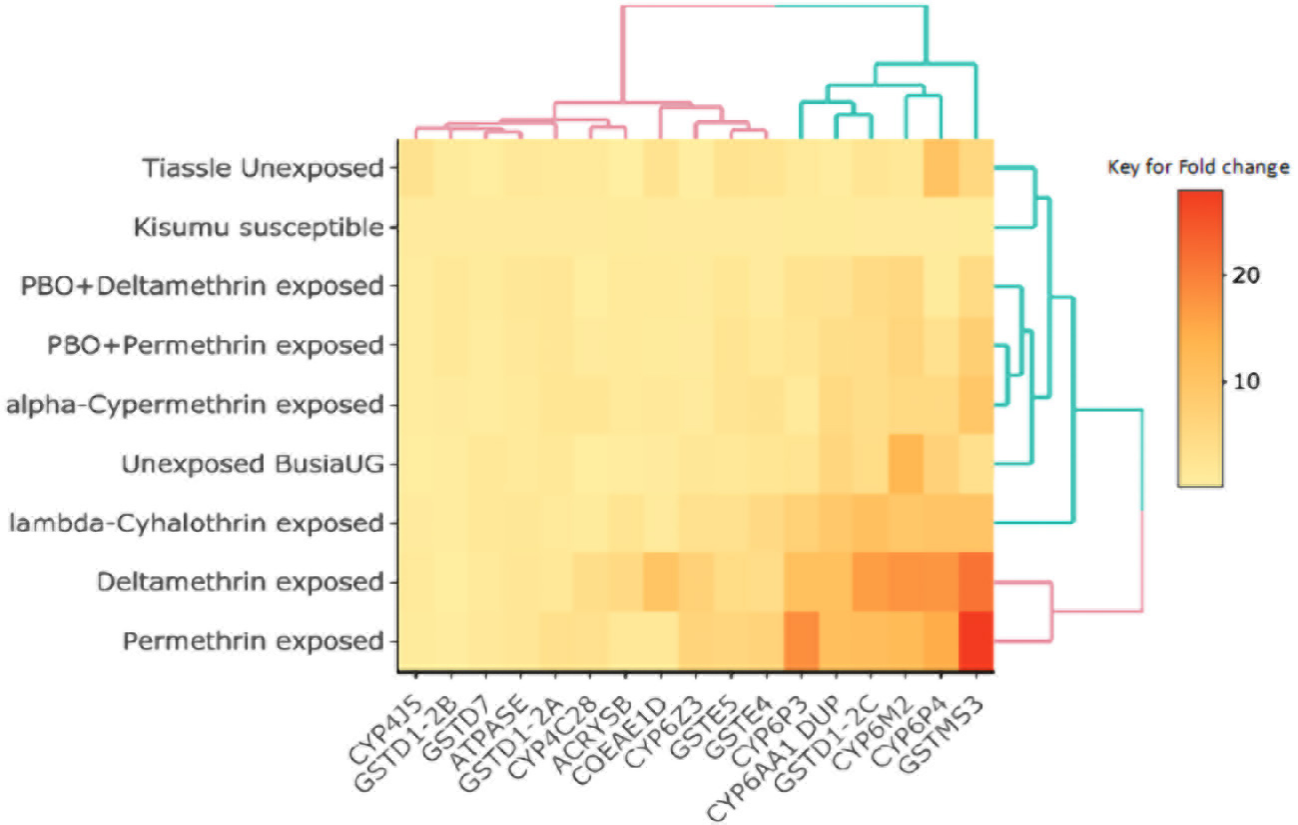
Heat map showing differential gene expression of the selected candidate genes from the different treatment groups relative to the susceptible control. The colours represent the fold change values with black being the lowest and red the highest. The heatmap was generated using R software (heatmaply package using k=2).

**Figure 3.**
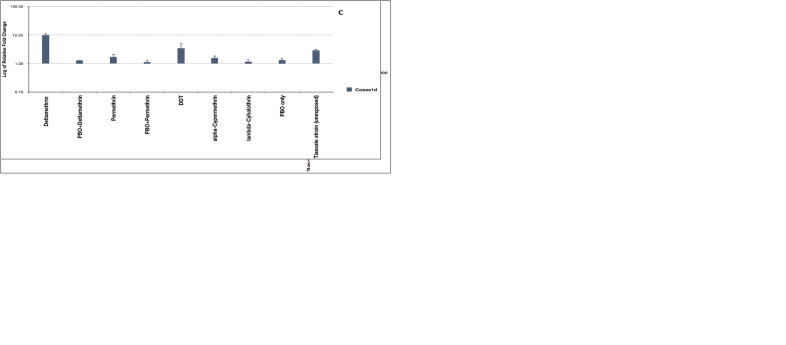
Relative fold change of gene expression of major gene families; GSTs, P450s and COEs, in treatment groups compared to unexposed Busia mosquitoes (resistant population). Transcription profiles of metabolic genes associated with insecticide resistance in Africa analysed by RT-qPCR shows unique expression patterns between mosquitoes exposed to PBO and non PBO. Mosquitoes pre-exposed to PBO only significantly overexpressed GSTMS3, GSTe5 and GSTD1 ( **A**) but down-regulated of all major P450s ( **B**). Mosquitoes exposed to only pyrethroids and DDT overexpressed almost all major genes including a COE, Coeae1d gene ( **C**) and genes for ATPase and alpha-crystalline proteins ( **D**). The west African resistant strain, Tiassale had a similar expression pattern for GSTs compared to BuisaUG colony though at lower levels but a different expression pattern for P450s. Data shows mean + SEM (error bars).

To examine differential gene expression, both exposed and unexposed mosquitoes were compared to susceptible mosquitoes, and we noted an upregulation of most genes from all the gene families studied with highest expression of GSTMS3 in permethrin survivors (mean fold change =27.98) ( Figure 2) (supplementary figure S1, Oruni, 2023c). To further examine if these genes were under induced or constitutive expression, we compared the exposed Busia mosquitoes with unexposed mosquitoes and of the 15 genes examined, 14 genes were overexpressed in mosquitoes that were exposed to the insecticides used in LLINs, deltamethrin and permethrin ( Figure 3A–D). All P450s showed evidence of induced expression although only CYP6P4 (14.19-17.49-fold), CYP6AA1 (11.16-11.44-fold), CYP6P3 (11.22-18.43-fold) and CYP6Z3 (6.4-7.05-fold) were significantly upregulated. The CYP4C28 gene was not considered because of low primer efficiency. Within the GST gene family (except GSTD7), all the genes – GSTMS3 (21.54-27.98-fold), GSTE4 (4.23-6.63-fold), GSTE5 (4.73-6.05-fold), GSTD1-exon 2C (11.77-16.71-fold) – were significantly upregulated following exposure. The expression patterns of CYP6M2, COEAE1D and GSTMS3 were less consistent where there was no significant difference in overexpression of CYP6M2 in exposed and unexposed mosquitoes (ANOVA; *F-stat* = 1.589, P = 0.276), COEAE1D was only significantly upregulated (10.19-fold, ANOVA; *F-stat* = 45.2899, P = 0.0025) in deltamethrin survivors, but not in those surviving exposure to other insecticides including other type 2 pyrethroids, alpha-cypermethrin and lambda-cyhalothrin, although exposure to alpha-cypermethrin and lambda-cyhalothrin caused a significant upregulation of only GSTMS3 (P<0.05). The GSTMS3 gene was significantly overexpressed in both non-PBO and PBO exposed mosquitoes. Strikingly, mosquitoes that were exposed to the synergist-PBO, showed that expression of GSTs was not significantly affected (ANOVA; F-statistic =2.13113, p=0.17001 for deltamethrin and ANOVA; F-statistic =2.11146, p=0.17185 for permethrin) compared to P450s where there was significant reduction in gene expression (ANOVA; F-statistic =10.30138, p=0.0075 for deltamethrin and ANOVA; F-statistic = 7.79701, p=0.01627 for permethrin) (supplementary Figure S1, Oruni, 2023c). When compared to unexposed BusiaUG, all P450s showed expression levels below the unexposed groups which was not seen in GSTs, COEs, ATPase and alpha-crystallin ( Figure 3A–B). The GSTs overexpressed in PBO-exposed mosquitoes, GSTMS3 (4.91-7.91-fold), GSTE5 (2.05-2.72-fold) and GSTD1 (4.17-4.49-fold), were significant in one or both PBO-pyrethroid exposed groups. However, GSTD7 and GSTE4 seemed to have been under-expressed after mosquitoes were exposed to PBO, similar to what was observed with the P450s. To understand this expression pattern, A STRING network analysis of the significantly overexpressed GSTs revealed multiple interaction networks including co-expression and similarity in protein homology (supplementary figure S3, Oruni, 2023c). The STRING analysis looks at both direct and indirect protein-protein interaction determined from both experiments and databases. The resultant outcome reveals whether proteins interact through co-expressions, gene fusions, occurrence, or all of them. The interpretation of this is whether proteins interact or just have similar pathways during gene expression.

A comparison of expression profiles between the unexposed resistant strains of BusiaUG and the Tiassale colony derived from West Africa, exhibited distinct transcriptomic profiles ( Figure 3C). Only seven out the 15 selected genes, that were all overexpressed in BusiaUG mosquitoes, were overexpressed in Tiassale and three out of the seven were significantly expressed, although at lower levels.

## Discussion

We detected very high levels of resistance to pyrethroids and DDT in Busia, eastern Uganda, with *Vgsc* kdr resistance nearly at fixation and moderate levels of the metabolic markers, *Cyp4J5* and *Coeae1d*, as has been previously reported ( Lynd *et al.*, 2019; Ojuka *et al.*, 2015). Resistance to the other type-2 pyrethroids, alpha-cypermethrin and lamda-cyhalothrin, which are not commonly used in Uganda, were less severe (50-60% mortality) compared to deltamethrin (<5% mortality). Although the suggested strategy would be recommending substitution of alpha-Cypermethrin or lambda-Cyhalothrin for permethrin in LLINs through the deployment of nets such as VEERALIN® and DuraNet®, recent evidence seems to suggest that this is not advisable as an IRM strategy since differences in susceptibility does not necessarily indicate operational relevance in performance unless the mode of action of the insecticide is completely different ( Lissenden *et al.*, 2021). Carbamates and organophosphates are still fully effective against *An. Gambiae* s.s. from Busia, and likely from eastern Uganda which is concordant with previous reports ( Mawejje *et al.,* 2013b; Okia *et al.,* 2013). The continued use of IRS in addition to PBO-LLINs may benefit vector control programs in the region as has been previously reported ( Katureebe *et al.*, 2016). The PBO-LLINs however, can only be used to complement IRS but not together. Recent evidence shows that combining IRS with PBO-LLINs can significantly reduce the effectiveness of the IRS with pirimiphos-methyl but not with bendiocarb in a resistant population of *An. Gambiae* ( Syme *et al.*, 2022). Nonetheless, although IRS programs coupled with LLINs may produce mixed results in some cases, usage of Actellic 300CS® (pirimiphos-methyl) has been quite effective in reducing malaria cases in Uganda ( Epstein *et al.*, 2022). However, the intense resistance levels are of concern for the continued usage of conventional LLINs (LLINs without PBO) that may only help prevent bites but not deliver mosquito lethality, thus providing only personal protection rather than community benefits ( Gleave *et al.*, 2021; Kleinschmidt *et al.*, 2015). Previous work showed that standard LLINs may not be sufficient to sustain malaria vector control even if supplemented by IRS using carbamates ( Katureebe *et al.*, 2016). On a positive note though, our results showed that exposing mosquitoes to a synergist, piperonyl butoxide (PBO), significantly increases mosquito mortality by pyrethroids which has also been reported previously ( Mawejje *et al.,* 2013b). Furthermore, we noted that deltamethrin PBO nets appear to be very effective against *An. gambiae* resistant populations and may therefore offer community protection. Our findings are in line with a recent cluster-randomized trial of PBO nets in Uganda, where malaria parasite prevalence in 2-10-year olds were significantly lower in areas where PBO nets were distributed compared to conventional LLINs after 18 months ( Staedke *et al.*, 2020) and 25 months (17.7% parasite prevalence in PBO-LLINs compared to 19.6% in Non PBO-LLINs, p=0.005) ( Maiteki-Sebuguzi *et al.*, 2023) post distribution. Maiteki-Sebuguzi *et al.* further showed that this reduction in malaria cases in PBO-LLINs areas might have been associated with reduction in vector density (542 mosquitoes collected in PBO-LLINs compared to 905 in Non PBO-LLINs, p<0.001). The effectiveness of PBO-LLINs in Uganda is reassuring, however, this does come with increased selection pressure from the mass distribution. It is possible that resistance to PBO nets could emerge and become widespread. In several studies reported by Gleave *et al*., analysis of mosquito mortality by PBO-LLINs suggests that this could already be happening elsewhere since the nets have been in use more frequently in some regions ( Gleave *et al.*, 2021). Using a time series analysis of the different studies, PBO and non PBO net efficacy was compared from experiments involving huts. In the analysis, studies were clustered into net type (PermaNet 3.0, Olyset Plus, PermaNet 2.0 and Olyset 2.0) and population resistance levels (high, moderate, low to susceptible). The results revealed the ability of PBO nets to kill mosquitoes decreased with time (from 2010 to 2018) for both PermaNet 3.0 and Olyset Plus in populations of *An. gambiae* mosquitoes that were highly resistant and only very effective in low to susceptible populations (supplementary figure S2, Oruni, 2023c). Nonetheless, PBO-LLINs still offered a better overall protective advantage compared to conventional LLINs (RR=1.63, 95% CI; 1.29-2.05, P<0.00001) ( Gleave *et al.*, 2021). However, all the studies considered in this analysis were from west Africa, which may not be comparable to the situation in east Africa given the differences in insecticide resistance profiles and intensity ( Barrimi *et al.*, 2013; Hancock *et al.*, 2020; Moyes *et al.*, 2020). We observed that the tolerance to PBO is more pronounced in combination with permethrin than deltamethrin as demonstrated in this research as well as previous studies ( Mawejje *et al.,* 2013b; Okia *et al.,* 2018), which is possibly due to the concentration of insecticide and PBO used in PermaNet compared to Olyset ( WHO, 2009; WHO, 2012).

Our analysis of gene expression showed that the intense pyrethroid resistance observed in Busia is likely driven by expression of multiple metabolic genes, majorly P450s like CYP6P4, CYP6AA1, CYP6P3, CYP6Z3. This is consistent with what has been previously reported in resistant African mosquito vectors ( Edi *et al.*, 2014; Ingham *et al.*, 2018; Irving *et al.*, 2012; Mulamba *et al.*, 2014b; Stica *et al.*, 2019b; Wilding *et al.*, 2014). Insecticide resistance in malaria vectors is known to be polygenic, where combinations of several genes are responsible for a resistance phenotype ( Ffrench-Constant, 2013). In most cases, the expression of the majority of these key genes conferring resistance are under induced expression while a few genes may be under constitutive expression ( Vontas *et al.*, 2005). For example, we noted that on top of the key P450s, the expression of some GSTs and COEs in Busia mosquitoes was induced, except for the CYP6M2 gene that had constitutive expression. This is consistent with what was reported by Djègbè *et al.* ( Djègbè *et al.*, 2014), who also showed that even increased exposure time did not increase the expression levels of CYP6M2.

Gene expression data from mosquitoes that survived PBO exposure indicated that there was induced overexpression of only GSTs such as of GSTMS3, GSTE5, GSTD1 and down regulation of the major P450s, compared to unexposed mosquitoes. Most of these GSTs are poorly characterized in *An. gambiae* resistance. Contrary to our findings, other studies have shown that exposure to PBO induced (rather than suppress) gene expression of P450s; for instance, in mice liver ( Watanabe *et al.*, 1998) and *Drosophila melanogaster* males ( Willoughby *et al.*, 2007). Besides the fact that there is a huge difference in complexity of systems between rodents and arthropods (pointing to different signaling pathways), none of the P450s reported in both studies were within the groups of sub-families implicated in insecticide resistance in malaria vectors. Hence, although PBO may increase gene expression of some P450s, there is currently no evidence that it induces the over-expression of any of the major genes involved in insecticide resistance as available data rather suggests the opposite ( Churcher *et al.*, 2016; Fadel *et al.*, 2019; Mawejje *et al.*, 2013b; Snoeck *et al.*, 2017). It is possible that the major P450s involved in insecticide resistance that have been selected over time, also happen to have an antagonistic relationship with PBO, although the exact reason why some P450s are downregulated while others are up-regulated remains to be investigated. In insects, PBO is largely known to combine with P450s and other oxidases forming a metabolite-inhibitory complex with enzyme, which effectively increases the potency of insecticides by blocking the metabolic activity of P450s or their isoenzymes that can detoxify the insecticide ( Farnham, 1999; Snoeck *et al.*, 2017). Therefore, mosquitoes that have alternative mechanisms or pathways like GSTs that can detoxify the insecticide but also evade the synergistic effects of PBO, could escape insecticide lethality in presence of PBO. Glutathione-s-transferases have been widely reported to be key enzymes strongly associated with insecticide resistance in African malaria vectors ( Hemingway *et al.*, 2004; Ingham *et al.,* 2018; Ranson *et al.*, 2000; Riveron *et al.*, 2014; Wilding *et al.*, 2015) and now possibly tolerance/resistance to PBO-pyrethroid combination. Our findings showed that possible resistance to PBO-pyrethroid combination may be mediated by significantly overexpressing closely related GSTs that also happen to interact with other major GSTs like GSTe2. These results therefore suggest that GSTs could be key genes in tolerance/resistance to PBO nets which might impact the efficacy of PBO nets in the future, as selection pressure mounts. Menze *et al* already showed that in *An. funestus*, a GST-mediated mechanism impacted PBO nets in Cameroon leading to loss of bed net efficacy ( Menze *et al.*, 2020). In our study, we analysed only 15 candidate genes by qPCR and therefore might have missed out on other key GSTs. We would recommend using RNAseq experiments to study PBO-pyrethroid exposed survivors in order to identify a full array of genes that are not affected by the synergistic effects of PBO and could also help identify possible novel markers which would be key in tracking PBO tolerance/resistance in real time. As a possible vector control strategy, incorporating diethyl maleate (DEM), an inhibitor of GSTs, ( Snoeck *et al.*, 2017) into mosaic LLINs could be an explorable option.

## Conclusion

We report that resistance to pyrethroids and DDT remains very high in eastern Uganda and is metabolically driven by multiple gene families. The use of LLINs without synergists therefore may not be very effective; PBO-LLINs, especially PermaNet 3.0, should possibly be the main area of focus for Uganda. However, finding mosquitoes that survive insecticides even in presence of PBO is worrying and could impact the efficiency of PBO-based nets in the future if or when resistance to PBO intensifies due to selection pressure. This study has demonstrated that glutathione-s-transferases could be majorly responsible for PBO-pyrethroid tolerance but a wider and more robust study is required to complement our findings. Meanwhile, a possibility of escalating tolerance/resistance to PBO nets should not be under looked; the mechanisms at play need to be studied in-depth and possible genetic markers for tracking its spread identified.

## Data Availability

figshare: qPCR Ct-values for the different groups of mosquitoes exposed and unexposed to insecticides. https://doi.org/10.6084/m9.figshare.22638916.v1 ( Oruni, 2023a). This project contains qPCR Ct-values extracted from AriaMx machine (Agilent© technologies) as a text report in Microsoft Excel® format. It includes Ct-values used to calculate the relative fold change for mosquito groups exposed and not exposed to insecticides. figshare: qPCR AriaMx files for the different groups of mosquitoes exposed and unexposed to insecticides. https://doi.org/10.6084/m9.figshare.22638964.v1 ( Oruni, 2023b). This project contains the AriaMx machine (Agilent© technologies) qPCR file which was used to extract the Ct-values ( Oruni, 2023a) into text report in Microsoft Excel® format. It can only be opened using the AriaMx software. figshare: 2022p TBD (Oruni *et al.*) v5_supplementary Figures.docx https://doi.org/10.6084/m9.figshare.22448395.v1 ( Oruni, 2023c). This project contains three figures: Figure S1- Transcription profiles of the most significantly overexpressed major genes studied in the different exposure groups; Figure S2- Time series analysis of An. gambiae mortality rates reveals decreasing ability of PBO nets to kill highly resistant mosquitoes; and Figure S3- STRING protein network analysis reveals interlinkage between the overexpressed genes in mosquitoes exposed to PBO. figshare: 2022p TBD (Oruni *et al.*) v5_supplementary table1.docx. https://doi.org/10.6084/m9.figshare.22448398.v1 ( Oruni, 2023d). This project contains a Table S1 showing the re-designed primers for genes used in the RT-qPCR for *Anopheles gambiae s.s.* Data are available under the terms of the Creative Commons Attribution 4.0 International license (CC-BY 4.0).

## References

[ref-1] Barrimi M Aalouane R Aarab C : Atlas on trends and current status of insecticide resistance in malaria vectors of the WHO African region. *Encephale.* 2013;53(1):59–65.

[ref-2] Bingham G Strode C Tran L : Can piperonyl butoxide enhance the efficacy of pyrethroids against pyrethroid-resistant *Aedes aegypti?* *Trop Med Int Health.* 2011;16(4):492–500. 10.1111/j.1365-3156.2010.02717.x 21324051

[ref-3] Chabi J Van’t Hof A N'dri LK : Rapid high throughput SYBR green assay for identifying the malaria vectors *Anopheles arabiensis, Anopheles coluzzii* and *Anopheles gambiae s.s. Giles*. *PLoS One.* 2019;14(4):e0215669. 10.1371/journal.pone.0215669 31002694 PMC6474623

[ref-4] Churcher TS Lissenden N Griffin JT : The impact of pyrethroid resistance on the efficacy and effectiveness of bednets for malaria control in Africa. *eLife.* 2016;5:e16090. 10.7554/eLife.16090 27547988 PMC5025277

[ref-5] David JP Ismail HM Chandor-Proust A : Role of cytochrome P450s in insecticide resistance: impact on the control of mosquito-borne diseases and use of insecticides on Earth. *Philos Trans R Soc Lond B Biol Sci.* 2013;368(1612):20120429. 10.1098/rstb.2012.0429 23297352 PMC3538419

[ref-6] Djègbè I Agossa FR Jones CM : Molecular characterization of DDT resistance in *Anopheles gambiae* from Benin. *Parasit Vectors.* 2014;7(1):409. 10.1186/1756-3305-7-409 25175167 PMC4164740

[ref-7] Djouaka R Riveron JM Yessoufou A : Multiple insecticide resistance in an infected population of the malaria vector *Anopheles funestus* in Benin. *Parasit Vectors.* 2016;9:453. 10.1186/s13071-016-1723-y 27531125 PMC4987972

[ref-8] Donnelly MJ Isaacs AT Weetman D : Identification, Validation, and Application of Molecular Diagnostics for Insecticide Resistance in Malaria Vectors. *Trends Parasitol.* 2016;32(3):197–206. 10.1016/j.pt.2015.12.001 26750864 PMC4767538

[ref-9] Edi CV Djogbénou L Jenkins AM : CYP6 P450 Enzymes and *ACE-1* Duplication Produce Extreme and Multiple Insecticide Resistance in the Malaria Mosquito *Anopheles gambiae*. *PLoS Genet.* 2014;10(3):e1004236. 10.1371/journal.pgen.1004236 24651294 PMC3961184

[ref-10] Epstein A Maiteki-Sebuguzi C Namuganga JF : Resurgence of malaria in Uganda despite sustained indoor residual spraying and repeated long lasting insecticidal net distributions. *PLOS Glob Public Health.* 2022;2(9):e0000676. 10.1371/journal.pgph.0000676 36962736 PMC10022262

[ref-11] Fadel AN Ibrahim SS Tchouakui M : A combination of metabolic resistance and high frequency of the 1014F *kdr* mutation is driving pyrethroid resistance in *Anopheles coluzzii* population from Guinea savanna of Cameroon. *Parasit Vectors.* 2019;12(1):263. 10.1186/s13071-019-3523-7 31133042 PMC6537440

[ref-12] Farnham AW : The Mode of Action of Piperonyl Butoxide with Reference to Studying Pesticide Resistance. *Piperonyl Butoxide.* 1999;199–213. 10.1016/B978-012286975-4/50014-0

[ref-13] Ffrench-Constant RH : The molecular genetics of insecticide resistance. *Genetics.* 2013;194(4):807–815. 10.1534/genetics.112.141895 23908373 PMC3730913

[ref-14] Gleave K Lissenden N Chaplin M : Piperonyl butoxide (PBO) combined with pyrethroids in insecticide- treated nets to prevent malaria in Africa (Review). 2021;5(5):CD012776. 10.1002/14651858.CD012776.pub3 PMC8142305 34027998

[ref-15] Hancock PA Hendriks CJM Tangena JA : Mapping trends in insecticide resistance phenotypes in African malaria vectors. *PLoS Biol.* 2020;18(6):e3000633. 10.1371/journal.pbio.3000633 32584814 PMC7316233

[ref-16] Hemingway J Hawkes NJ McCarroll L : The molecular basis of insecticide resistance in mosquitoes. *Insect Biochem Mol Biol.* 2004;34(7):653–665. 10.1016/j.ibmb.2004.03.018 15242706

[ref-17] Ingham VA Wagstaff S Ranson H : Transcriptomic meta-signatures identified in *Anopheles gambiae* populations reveal previously undetected insecticide resistance mechanisms. *Nat Commun.* 2018;9(1):5282. 10.1038/s41467-018-07615-x 30538253 PMC6290077

[ref-18] Irving H Riveron JM Ibrahim SS : Positional cloning of rp2 QTL associates the P450 genes *CYP6Z1, CYP6Z3* and *CYP6M7* with pyrethroid resistance in the malaria vector *Anopheles funestus*. *Heredity (Edinb).* 2012;109(6):383–392. 10.1038/hdy.2012.53 22948188 PMC3499844

[ref-19] Katureebe A Zinszer K Arinaitwe E : Measures of Malaria Burden after Long-Lasting Insecticidal Net Distribution and Indoor Residual Spraying at Three Sites in Uganda: A Prospective Observational Study. *PLoS Med.* 2016;13(11):e1002167. 10.1371/journal.pmed.1002167 27824885 PMC5100985

[ref-20] Kleinschmidt I Mnzava AP Kafy HT : Design of a study to determine the impact of insecticide resistance on malaria vector control: a multi-country investigation. *Malar J.* 2015;14:282. 10.1186/s12936-015-0782-4 26194648 PMC4508808

[ref-21] Lissenden N Kont MD Essandoh J : Review and meta-analysis of the evidence for choosing between specific pyrethroids for programmatic purposes. *Insects.* 2021;12(9):826. 10.3390/insects12090826 34564266 PMC8465213

[ref-22] Lynd A Gonahasa S Staedke SG : LLIN Evaluation in Uganda Project (LLINEUP): A cross-sectional survey of species diversity and insecticide resistance in 48 districts of Uganda. *Parasit Vectors.* 2019;12(1):94. 10.1186/s13071-019-3353-7 30867018 PMC6417037

[ref-23] Lynd A Oruni A Van’T Hof AE : Insecticide resistance in *Anopheles gambiae* from the northern Democratic Republic of Congo, with extreme knockdown resistance ( *kdr*) mutation frequencies revealed by a new diagnostic assay. *Malar J.* 2018;17(1):412. 10.1186/s12936-018-2561-5 30400885 PMC6219172

[ref-24] Maiteki-Sebuguzi C Gonahasa S Kamya MR : Effect of long-lasting insecticidal nets with and without piperonyl butoxide on malaria indicators in Uganda (LLINEUP): final results of a cluster-randomised trial embedded in a national distribution campaign. *Lancet Infect Dis.* 2023;23(2):247–258. 10.1016/S1473-3099(22)00469-8 36174592

[ref-25] Mawejje HD Wilding CS Rippon EJ : Insecticide resistance monitoring of field-collected *anopheles gambiae s.l.* populations from jinja, eastern uganda, identifies high levels of pyrethroid resistance. *Med Vet Entomol.* 2013a;27(3):276–83. 10.1111/j.1365-2915.2012.01055.x 23046446 PMC3543752

[ref-26] Mawejje HD Wilding CS Rippon EJ : Insecticide resistance monitoring of field-collected Anopheles gambiae s.l. populations from Jinja, eastern Uganda, identifies high levels of pyrethroid resistance. *Med Vet Entomol.* 2013b;44(3):735–745. 10.1111/j.1365-2915.2012.01055.x PMC3543752 23046446

[ref-27] Menze BD Kouamo MF Wondji MJ : An Experimental Hut Evaluation of PBO-Based and Pyrethroid-Only Nets against the Malaria Vector *Anopheles funestus* Reveals a Loss of Bed Nets Efficacy Associated with *GSTe2* Metabolic Resistance. *Genes (Basel).* 2020;11(2):143. 10.3390/genes11020143 32013227 PMC7073577

[ref-29] Morgan JC Irving H Okedi LM : Pyrethroid Resistance in an *Anopheles funestus* Population from Uganda. *PLoS One.* 2010;5(7):e11872. 10.1371/journal.pone.0011872 20686697 PMC2912372

[ref-30] Moyes CL Athinya DK Seethaler T : Evaluating insecticide resistance across african districts to aid malaria control decisions. *Proc Natl Acad Sci U S A.* 2020;117(36):22042–22050. 10.1073/pnas.2006781117 32843339 PMC7486715

[ref-31] Mugenzi LMJ Menze BD Tchouakui M : *Cis*-regulatory *CYP6P9b* P450 variants associated with loss of insecticide-treated bed net efficacy against *Anopheles funestus.* *Nat Commun.* 2019;10(1):4652. 10.1038/s41467-019-12686-5 31604938 PMC6789023

[ref-32] Mulamba C Irving H Riveron JM : Contrasting *Plasmodium* infection rates and insecticide susceptibility profiles between the sympatric sibling species *Anopheles parensis* and *Anopheles funestus s.s:* a potential challenge for malaria vector control in Uganda. *Parasit Vectors.* 2014a;7:71. 10.1186/1756-3305-7-71 24533773 PMC3937429

[ref-33] Mulamba C Riveron JM Ibrahim SS : Widespread pyrethroid and DDT resistance in the major malaria vector *anopheles funestus* in East Africa is driven by metabolic resistance mechanisms. *PLoS One.* 2014b;9(10):e110058. 10.1371/journal.pone.0110058 25333491 PMC4198208

[ref-34] Njoroge H van’t Hof A Oruni A : Identification of a rapidly-spreading triple mutant for high-level metabolic insecticide resistance in *Anopheles gambiae* provides a real-time molecular diagnostic for antimalarial intervention deployment. *Mol Ecol.* 2022;31(16):4307–4318. 10.1111/mec.16591 35775282 PMC9424592

[ref-35] Ojuka P Boum Y 2nd Denoeud-Ndam L : Early biting and insecticide resistance in the malaria vector Anopheles might compromise the effectiveness of vector control intervention in Southwestern Uganda. *Malar J.* 2015;14:148. 10.1186/s12936-015-0653-z 25879539 PMC4416237

[ref-36] Okia M Hoel DF Kirunda J : Insecticide resistance status of the malaria mosquitoes: *Anopheles gambiae* and *Anopheles funestus* in eastern and northern Uganda. *Malar J.* 2018;17(1):157. 10.1186/s12936-018-2293-6 29625585 PMC5889576

[ref-37] Okia M Ndyomugyenyi R Kirunda J : Bioefficacy of long-lasting insecticidal nets against pyrethroid-resistant populations of *Anopheles gambiae s.s.* from different malaria transmission zones in Uganda. *Parasit Vectors.* 2013;6:130. 10.1186/1756-3305-6-130 23634798 PMC3656772

[ref-38] Oruni A : qPCR Ct-values for the different groups of mosquitoes exposed and unexposed to insecticides. figshare. [Dataset],2023a. 10.6084/m9.figshare.22638916.v1

[ref-39] Oruni A : qPCR AriaMx files for the different groups of mosquitoes exposed and unexposed to insecticides. figshare. [Dataset],2023b. 10.6084/m9.figshare.22638964.v1

[ref-40] Oruni A : 2022p TBD (Oruni et al) v5_supplementary Figures.docx. figshare. Figure,2023c. 10.6084/m9.figshare.22448395.v1

[ref-41] Oruni A : 2022p TBD (Oruni et al) v5_supplementary table1.docx. figshare.Dataset. 2023d. 10.6084/m9.figshare.22448398.v1

[ref-28] Pfaffl MW : Mathematical modelling of prefermenters - I. Model development and verification. *Water Research.* 1999;33(12):2757–2768.

[ref-42] Pondeville E David JP Guittard E : Microarray and RNAi analysis of P450s in *Anopheles gambiae* male and female steroidogenic tissues: *CYP307A1* is required for ecdysteroid synthesis. *PLoS One.* 2013;8(12):e79861. 10.1371/journal.pone.0079861 24324583 PMC3851169

[ref-43] Ranson H Jensen B Wang X : Genetic mapping of two loci affecting DDT resistance in the malaria vector *Anopheles gambiae*. *Insect Mol Biol.* 2000;9(5):499–507. 10.1046/j.1365-2583.2000.00214.x 11029668

[ref-44] Ranson H Lissenden N : Insecticide Resistance in African *Anopheles* Mosquitoes: A Worsening Situation that Needs Urgent Action to Maintain Malaria Control. *Trends Parasitol.* 2016;32(3):187–196. 10.1016/j.pt.2015.11.010 26826784

[ref-45] Riveron JM Yunta C Ibrahim SS : A single mutation in the *GSTe2* gene allows tracking of metabolically based insecticide resistance in a major malaria vector. *Genome Biol.* 2014;15(2):R27. 10.1186/gb-2014-15-2-r27 24565444 PMC4054843

[ref-46] Rugnao S Gonahasa S Maiteki-Sebuguzi C : LLIN Evaluation in Uganda Project (LLINEUP): Factors associated with childhood parasitaemia and anaemia 3 years after a national long-lasting insecticidal net distribution campaign: A cross-sectional survey. *Malar J.* 2019;18(1):207. 10.1186/s12936-019-2838-3 31234882 PMC6591906

[ref-47] Schmittgen TD Livak KJ : Analyzing real-time PCR data by the comparative *C* _T_ method. *Nat Protoc.* 2008;3(6):1101–1108. 10.1038/nprot.2008.73 18546601

[ref-48] Scott JG : Cytochromes P450 and insecticide resistance. *Insect Biochem Mol Biol.* 1999;29(9):757–777. 10.1016/s0965-1748(99)00038-7 10510498

[ref-49] Snoeck S Greenhalgh R Tirry L : The effect of insecticide synergist treatment on genome-wide gene expression in a polyphagous pest. *Sci Rep.* 2017;7(1):13440. 10.1038/s41598-017-13397-x 29044179 PMC5647426

[ref-50] Staedke SG Gonahasa S Dorsey G : Effect of long-lasting insecticidal nets with and without piperonyl butoxide on malaria indicators in Uganda (LLINEUP): a pragmatic, cluster-randomised trial embedded in a national LLIN distribution campaign. *Lancet.* 2020;395(10232):1292–1303. 10.1016/S0140-6736(20)30214-2 32305094 PMC7181182

[ref-51] Stica C Jeffries CL Irish SR : Characterizing the molecular and metabolic mechanisms of insecticide resistance in *Anopheles gambiae* in Faranah, Guinea. *Malar J.* 2019a;18(1):244. 10.1186/s12936-019-2875-y 31315630 PMC6637595

[ref-52] Stica C Jeffries CL Irish SR : Characterizing the molecular and metabolic mechanisms of insecticide resistance in *Anopheles gambiae* in Faranah, Guinea. *Malar J.* 2019b;18(1):244. 10.1186/s12936-019-2875-y 31315630 PMC6637595

[ref-53] Syme T Gbegbo M Obuobi D : Pyrethroid-piperonyl butoxide (PBO) nets reduce the efficacy of indoor residual spraying with pirimiphos-methyl against pyrethroid-resistant malaria vectors. *Sci Rep.* 2022;12(1):6857. 10.1038/s41598-022-10953-y 35478216 PMC9046380

[ref-54] Szklarczyk D Gable AL Lyon D : STRING v11: protein-protein association networks with increased coverage, supporting functional discovery in genome-wide experimental datasets. *Nucleic Acids Res.* 2019;47(D1):D607–D613. 10.1093/nar/gky1131 30476243 PMC6323986

[ref-55] The *Anopheles gambiae* 1000 Genomes Consortium, Data analysis group, Partner working group, *et al.* : Genetic diversity of the African malaria vector *Anopheles gambiae*. *Nature.* 2017;552(7683):96–100. 10.1038/nature24995 29186111 PMC6026373

[ref-56] Vontas J Blass C Koutsos AC : Gene expression in insecticide resistant and susceptible *Anopheles gambiae* strains constitutively or after insecticide exposure. *Insect Mol Biol.* 2005;14(5):509–521. 10.1111/j.1365-2583.2005.00582.x 16164607

[ref-57] Watanabe T Manabe S Ohashi Y : Comparison of the Induction Profile of Hepatic Drug-metabolising Enzymes Between Piperonyl Butoxide and Phenobarbital in Rats. *J Toxicol Pathol.* 1998;11(1):1–10. 10.1293/tox.11.1

[ref-58] Weetman D Wilding CS Neafsey DE : Candidate-gene based GWAS identifies reproducible DNA markers for metabolic pyrethroid resistance from standing genetic variation in East African *Anopheles gambiae*. *Sci Rep.* 2018;8(1):2920. 10.1038/s41598-018-21265-5 29440767 PMC5811533

[ref-59] WHO: Report of the twelfth WHOPES working group meeting, WHO/HQ, Geneva, 8-11 December 2008. 2009;120. Reference Source

[ref-60] WHO: Report of the fifteenth WHOPES working group meeting: WHO/HQ, Geneva, 18- 22 June 2012 Review of Olyset plus, Interceptor LN Malathion 440 EW Vectobac GR. 2012;4–20. Reference Source

[ref-61] WHO: IMPLICATIONS OF INSECTICIDE RESISTANCE ON MALARIA VECTOR CONTROL Implications of Insecticide Resistance on Malaria Vector Control Project. February 2014, 2016.

[ref-62] WHO: The evaluation process for vector control products.Who/Htm/Gmp/2017.13, June. 2017;10. Reference Source

[ref-63] WHO: World malaria report 2018. 2018. Reference Source

[ref-64] WHO/GMP: Test procedures for insecticide resistance monitoring in malaria vector mosquitoes: Second edition. World Health Organisation Technical Report Series,2016;22. Reference Source

[ref-65] Wilding CS Weetman D Rippon EJ : Parallel evolution or purifying selection, not introgression, explain similarity in the pyrethroid detoxification linked GSTE4 of *Anopheles gambiae* and *An. arabiensis*. 2015;290(1):201–215. 10.1007/s00438-014-0910-9 PMC4312195 25213601

[ref-66] Wilding CS Weetman D Rippon EJ : Parallel evolution or purifying selection, not introgression, explains similarity in the pyrethroid detoxification linked GSTE4 of *Anopheles gambiae* and *An. arabiensis*. *Mol Genet Genomics.* 2014;290(1):201–215. 10.1007/s00438-014-0910-9 25213601 PMC4312195

[ref-67] Williams J Flood L Praulins G : Characterisation of *Anopheles* strains used for laboratory screening of new vector control products. *Parasit Vectors.* 2019;12(1):522. 10.1186/s13071-019-3774-3 31690332 PMC6833243

[ref-68] Willoughby L Batterham P Daborn PJ : Piperonyl butoxide induces the expression of cytochrome P450 and glutathione S-transferase genes in *Drosophila melanogaster*. *Pest Manag Sci.* 2007;63(8):803–808. 10.1002/ps.1391 17514638

[ref-69] Wondji CS Irving H Morgan J : Two duplicated P450 genes are associated with pyrethroid resistance in *Anopheles funestus*, a major malaria vector. *Genome Res.* 2009;19(3):452–459. 10.1101/gr.087916.108 19196725 PMC2661802

