# Supplementary material for: Pyrethroid resistance and gene expression profile of a new resistant An. gambiae colony from Uganda reveals multiple resistance mechanisms and overexpression of Glutathione-S-Transferases linked to survival of PBO-pyrethroid combination
Source: Wellcome Open Res. 2024 Apr 29;9:13. Originally published 2024 Jan 8. [Version 2] doi: 10.12688/wellcomeopenres.19404.2 (PMC11134160; doi:10.12688/wellcomeopenres.19404.2)
Supplement: Peer review history

# Reviewer report: Reviewer response for version 2

**DOI:** 10.21956/wellcomeopenres.23659.r81002

**Published:** 2024-05-28

**Authors:** Salum Azizi

**Affiliations:** 1 Kilimanjaro Christian Medical University College, Moshi, Tanzania

I confirm that all the queries that I raised in the review have now been sufficiently addressed.

Is the work clearly and accurately presented and does it cite the current literature?

Yes

If applicable, is the statistical analysis and its interpretation appropriate?

I cannot comment. A qualified statistician is required.

Are all the source data underlying the results available to ensure full reproducibility?

Partly

Is the study design appropriate and is the work technically sound?

Yes

Are the conclusions drawn adequately supported by the results?

Yes

Are sufficient details of methods and analysis provided to allow replication by others?

Partly

Reviewer Expertise:

Medical Entomologist, evaluation of new mosquito control products (insecticide-based)

I confirm that I have read this submission and believe that I have an appropriate level of expertise to confirm that it is of an acceptable scientific standard.

# Reviewer report: Reviewer response for version 2

**DOI:** 10.21956/wellcomeopenres.23659.r81001

**Published:** 2024-05-16

**Authors:** Ahmed Idowu Omotayo

**Affiliations:** 1 Lagos State University of Science and Technology, Ikorodu, Lagos, Nigeria

The concerns I raised with the first version have been adequately addressed.

Is the work clearly and accurately presented and does it cite the current literature?

Yes

If applicable, is the statistical analysis and its interpretation appropriate?

Yes

Are all the source data underlying the results available to ensure full reproducibility?

Yes

Is the study design appropriate and is the work technically sound?

Yes

Are the conclusions drawn adequately supported by the results?

Yes

Are sufficient details of methods and analysis provided to allow replication by others?

Yes

Reviewer Expertise:

Vector Biology

I confirm that I have read this submission and believe that I have an appropriate level of expertise to confirm that it is of an acceptable scientific standard.

# Reviewer report: Reviewer response for version 1

**DOI:** 10.21956/wellcomeopenres.21493.r72579

**Published:** 2024-02-26

**Authors:** Ahmed Idowu Omotayo

**Affiliations:** 1 Lagos State University of Science and Technology, Ikorodu, Lagos, Nigeria

The study set out to contribute to our knowledge of insecticide resistance mechanisms in wild populations of malaria vectors especially in Uganda. It also seeks to assess the efficacy and appropriateness of PBO-LLINs for the control of malaria vectors. 

 They design is great and well planned and the whole study is excellent, but I have few comments and questions.

 MAJOR COMMENT

 1.) The natural level resistance profile of the BusiaUG, perhaps at G1 is absent except for deltamethrin, any reason for that? Having the resistance profile for all the insecticides from G1 would have been good for comparison and to understand the baseline profile of the colony. I think the issue might be the population from G1 would not be enough to run that, if yes, adding a sentence about that will be good but if No, kindly explain.

 MINOR COMMENT

 Introduction

 1.) The references about association of cytochrome P450 enzymes to pyrethroid resistance has references 10 years upwards. I am of the opinion that there are more recent studies also on this and that would be better to reference. If possible, give recent publications 2019 to 2024 and the references more than 10 years can be deleted. Authors such as Etang, Edi, Omotayo Mohammed and others have more recent publications on this.

 2.) "There are reports of mosquitoes surviving PBO-pyrethroid exposure from a number of countries in Africa including Uganda ( Gleave *et al.*, 2021)".

 I think there should be a comma after this sentence.

 Methods

 1.) Hope the "arm feeding" method of female adult mosquitoes was properly approved with the ethical approval?

 2.) Under the "WHO bioassay (resistance phenotyping)", the G1 exposed to discriminating dose of deltamethrin, was it the standard WHO insecticide impregnated papers that was used as used for the G9-G11 exposure? If yes, let it be clear that the standard WHO insecticide impregnated papers were used and if not, please describe how the exposure to discriminating dose was done. I am guessing the exposure papers if not standard WHO papers was done be the investigators.

 3.) Under the "WHO bioassay (resistance phenotyping)", the 5-10 pools of alive mosquitoes for each insecticide type for gene expression analysis, whats the number of mosquitoes in each pool? Can we add it. If you mean 5-10 mosquitoes were polled together, can we make the sentence clearer?

 I think the conclusion is very appropriate and the recommendation is good.

Is the work clearly and accurately presented and does it cite the current literature?

Yes

If applicable, is the statistical analysis and its interpretation appropriate?

Yes

Are all the source data underlying the results available to ensure full reproducibility?

Yes

Is the study design appropriate and is the work technically sound?

Yes

Are the conclusions drawn adequately supported by the results?

Yes

Are sufficient details of methods and analysis provided to allow replication by others?

Yes

Reviewer Expertise:

Vector Biology

I confirm that I have read this submission and believe that I have an appropriate level of expertise to confirm that it is of an acceptable scientific standard, however I have significant reservations, as outlined above.

# Response

**Published:** 2024-04-04

**Authors:** Ambrose Oruni

**Affiliations:** Makerere University, Kampala, Central Region, Uganda

1. The study set out to contribute to our knowledge of insecticide resistance mechanisms in wild populations of malaria vectors especially in Uganda. It also seeks to assess the efficacy and appropriateness of PBO-LLINs for the control of malaria vectors. They design is great and well planned and the whole study is excellent, but I have few comments and questions. **Response;** We appreciate the reviewer for the kind complements. 2. MAJOR COMMENT 1.) The natural level resistance profile of the BusiaUG, perhaps at G1 is absent except for deltamethrin, any reason for that? Having the resistance profile for all the insecticides from G1 would have been good for comparison and to understand the baseline profile of the colony. I think the issue might be the population from G1 would not be enough to run that, if yes, adding a sentence about that will be good but if No, kindly explain. **Response;** we only captured this at F1 using pyrethroid and PBO nets as indicated in the edited text. We could only access WHO cone assays in the field for use to ascertain the resistance profile of the population. Indeed, we would have liked to conduct a full insecticide resistance profiling but we didn’t do this because our major aim was to maximize the G1 numbers to increase the chances of successfully establishing a colony. We therefore used only deltamethrin to give us an idea of how resistant G1 population is. I have added a sentence as suggested to explain this. 3. MINOR COMMENT Introduction 1.) The references about association of cytochrome P450 enzymes to pyrethroid resistance has references 10 years upwards. I am of the opinion that there are more recent studies also on this and that would be better to reference. If possible, give recent publications 2019 to 2024 and the references more than 10 years can be deleted. Authors such as Etang, Edi, Omotayo Mohammed and others have more recent publications on this. **Response; **I think this shouldn’t be a problem. Some of the references mentioned that are above 10 years were articles giving a detailed discussion of P450s, other were discovery of P450s in a different Anopheles species and most of the recent articles still refer to some of these earlier papers. We also wanted to capture the timeline in relation to contribution of P450s to insecticide resistance given that temporal changes have been evident. 2.) "There are reports of mosquitoes surviving PBO-pyrethroid exposure from a number of countries in Africa including Uganda (Gleave et al., 2021)". I think there should be a comma after this sentence. **Response;** we appreciate this observation; this has been corrected. 4. Methods 1.) Hope the "arm feeding" method of female adult mosquitoes was properly approved with the ethical approval? **Response; **Yes, we got all ethical approvals to conduct this study. 2.) Under the "WHO bioassay (resistance phenotyping)", the G1 exposed to discriminating dose of deltamethrin, was it the standard WHO insecticide impregnated papers that was used as used for the G9-G11 exposure? If yes, let it be clear that the standard WHO insecticide impregnated papers were used and if not, please describe how the exposure to discriminating dose was done. I am guessing the exposure papers if not standard WHO papers was done be the investigators. **Response;** we appreciate this comment and to clarify further, Standard WHO papers were used for 1x, 5x, and 10 diagnostic doses since they are available on market. However, we prepared the papers for 2x, 3x diagnostic doses using WHO protocol. I have added a statement to reflect this. 3.) Under the "WHO bioassay (resistance phenotyping)", the 5-10 pools of alive mosquitoes for each insecticide type for gene expression analysis, whats the number of mosquitoes in each pool? Can we add it. If you mean 5-10 mosquitoes were polled together, can we make the sentence clearer? **Response;** it is pools of 5-10 mosquitoes. To explain this, each pool is a biological replicate as indicated in the texts. I have clarified this. 5. I think the conclusion is very appropriate and the recommendation is good. **Response;** we appreciate the reviewer for the kind words.

# Reviewer report: Reviewer response for version 1

**DOI:** 10.21956/wellcomeopenres.21493.r72587

**Published:** 2024-02-01

**Authors:** Salum Azizi

**Affiliations:** 1 Kilimanjaro Christian Medical University College, Moshi, Tanzania

1. The observed decrease in egg laying from G1 to G6 was responded by increasing frequency of blood feeding, what was the response? did it restored the egg laying as in G1?

 2. Field mosquito collection explained here is for the 384 mosquitoes used for the establishment of the colony (BusiaUG), but information on where and how wild mosquitoes that was compared with BusiaUG in the phenotypic resistance profiling is not explained

 3. Generation 12 of BusiaUG was selected using 0.05% Deltamethrin, what was it for?

 4. Figure 1 is not clear in the following aspects: Wild, G1 and G9 were tested using different bioassays (cone bioassays using bed nets tested against Busia wild, deltamethrin resistance intensity tube bioassays tested against G1, and  a range of insecticides at diagnostic concentration with or without synergist at G9) making it difficult to compare the results between wild and colony mosquitoes. Wild mosquitoes might be close to G1, however G9 is a distant generation from a wild population (it might already been affected with laboratory conditions in the insectary, in terms of fitness, you have observed a decrease of egg laying for example from G1 to G6), having a wild colony as a comparator in panel B and C is therefore important. Is panel A results obtained at the same time as panel B or C?

 5. Information on mosquito sampling and preservation for transcription studies is not provided. It is only mentioned that exposed and unexposed mosquitoes were compared to susceptible mosquitoes, but it is not clear how many were taken and whether survivals were separated from the ones which died among the exposed.

 6. Some text in Fig 3 are not readable (improve size/readability)

 7. The title of your manuscript is centred on "a new resistant An. gambiae colony from Uganda", but the discussion is mainly focused on wild mosquitoes from Busia. Even the introduction lacks justification why in the first place the "new resistant colony" establishment was important.

Is the work clearly and accurately presented and does it cite the current literature?

Yes

If applicable, is the statistical analysis and its interpretation appropriate?

I cannot comment. A qualified statistician is required.

Are all the source data underlying the results available to ensure full reproducibility?

Partly

Is the study design appropriate and is the work technically sound?

Yes

Are the conclusions drawn adequately supported by the results?

Yes

Are sufficient details of methods and analysis provided to allow replication by others?

Partly

Reviewer Expertise:

Medical Entomologist, evaluation of new mosquito control products (insecticide-based)

I confirm that I have read this submission and believe that I have an appropriate level of expertise to confirm that it is of an acceptable scientific standard, however I have significant reservations, as outlined above.

# Response

**Published:** 2024-04-04

**Authors:** Ambrose Oruni

**Affiliations:** Makerere University, Kampala, Central Region, Uganda

1. The observed decrease in egg laying from G1 to G6 was responded by increasing frequency of blood feeding, what was the response? did it restored the egg laying as in G1? **Response;** It didn't restore the egg-laying, it made sure that the colony didn’t collapse by increasing the number of blood fed mosquitoes hence increasing the chances of obtaining eggs and more adults in the next generation. 2. Field mosquito collection explained here is for the 384 mosquitoes used for the establishment of the colony (BusiaUG), but information on where and how wild mosquitoes that was compared with BusiaUG in the phenotypic resistance profiling is not explained **Response; **The wild  mosquitoes were collected from the same area using the same technique during a baseline survey to determine if the area was viable for collecting mosquitoes for colony establishment especially since IRS was done in the neighboring district. After this collection, we also took advantage to assess the baseline resistance profile. Unfortunately, we didn’t take note of how many mosquitoes were collected during the baseline. I have made a notice of this. 3. Generation 12 of BusiaUG was selected using 0.05% Deltamethrin, what was it for? **Response;** This was to maintain resistance within the colony into the subsequent generations. 4. Figure 1 is not clear in the following aspects: Wild, G1 and G9 were tested using different bioassays (cone bioassays using bed nets tested against Busia wild, deltamethrin resistance intensity tube bioassays tested against G1, and  a range of insecticides at diagnostic concentration with or without synergist at G9) making it difficult to compare the results between wild and colony mosquitoes. Wild mosquitoes might be close to G1, however G9 is a distant generation from a wild population (it might already been affected with laboratory conditions in the insectary, in terms of fitness, you have observed a decrease of egg laying for example from G1 to G6), having a wild colony as a comparator in panel B and C is therefore important. Is panel A results obtained at the same time as panel B or C? **Response;** I have made some clarification of this. We collected wild mosquitoes at baseline, reared F1s in Uganda and then used WHO cone assays (because that’s what was available) to measure the resistance of the wild population. Indeed, at G1, we performed bioassays using deltamethrin response curve only because we didn’t want to use too many mosquitoes for colony establishment. The assumption was that there was no significant difference in the resistance profile from wild and G1. We then performed an array of insecticide phenotyping at G9 when we had a colony and a surplus of adults to use. We checked if; the colony hadn’t lost resistance (which they didn’t) and profile to other insecticides. We couldn’t compare with wild because colony establishment was done in Liverpool hence, we couldn’t collect more wild mosquitoes. Panel A results were obtained at baseline and panel B was obtained at G1 (about 2 weeks after collection) and panel C were obtained after colony establishment (about 6 months after collection). This has been explained in the figure legend. 5. Information on mosquito sampling and preservation for transcription studies is not provided. It is only mentioned that exposed and unexposed mosquitoes were compared to susceptible mosquitoes, but it is not clear how many were taken and whether survivals were separated from the ones which died among the exposed. **Response;** We mention this under RT-qPCR sub heading in the first paragraph explaining the RNA extraction. We used biological replicates (pools) containing 5-10 mosquitoes. I have clarified this in the text that we only used mosquitoes that survived the exposure i.e. alive. 6. Some text in Fig 3 are not readable (improve size/readability) **Response;** I have submitted a clearer new figure. 7. The title of your manuscript is centred on "a new resistant An. gambiae colony from Uganda", but the discussion is mainly focused on wild mosquitoes from Busia. Even the introduction lacks justification why in the first place the "new resistant colony" establishment was important. **Response1;** The new colony was established from the same wild population with characteristics and resistance profile that was very similar to the wild/field population given the similarity in pyrethroid resistance level at G9-G11. Hence, the colony was used as foundation to understand the resistance patterns in the *An. gambaie* mosquitoes from Busia. **Response 2; **We didn’t justify why the colony was established because it was part of a bigger study of the main grant and we simply took advantage to perform additional experiments given the opportunity. The justification was for why we performed the experiments, which I think is clearly stipulated.
